# Supplementary material for: Are carbon emissions trading and green financial instruments synergistic? -Comprehensive quantitative research based on content analysis
Source: PLoS One. 2024 Mar 7;19(3):e0298601. doi: 10.1371/journal.pone.0298601 (PMC10919601; doi:10.1371/journal.pone.0298601)
Supplement: S1 Data — (ZIP) [file pone.0298601.s001.zip › Data/地级市Z_F工作报告.zip!/╜¡╦╒/│ú╓▌╩╨/└┤╫╘╣1⁄2╓┌║┼í╛┬φ┐╦╩2╛▌═°í┐_2004.docx]

各位代表：

　　现在，我代表常州市人民政府，向大会作工作报告，请予审议，并请市政协各位委员和其他列席人员提出意见。

　　一、2003年工作回顾

　　去年是本届政府任期的第一年，也是常州经济社会事业取得重大进展的一年。一年来，全市人民在市委的正确领导下，以邓小平理论和“三个代表”重要思想为指导，认真贯彻落实党的十六大精神，迎难而上，艰苦奋斗，开拓进取，扎实工作，圆满地完成了市十三届人大一次会议确定的各项任务。

　　——国民经济跃上新台阶。全市人均国内生产总值首次突破3000美元，标志着我市国民经济步入了新的重要发展期。全年完成国内生产总值900.2亿元，按可比价增长14.5%。实现财政收入136.1亿元，增长32.3%。完成全社会固定资产投资446.6亿元，增长78.6%。工业保持“高投入、快增长”的良好势头。规模以上工业企业实现增加值366.5亿元，增长31%，实现利税112.3亿元，增长30.5%，工业投入完成250.8亿元，增长102.9%；大企业大集团建设加快推进，常林集团、新科集团销售收入分别突破70亿元和60亿元，中天钢铁公司上缴税收超亿元，新科激光视盘机、红联室内灯具荣获“中国名牌”称号；高新技术产业产值占全部工业总产值的26%，比去年提高1.4个百分点。农村经济结构调整步伐加快，农业产业化龙头企业带动作用明显增强，农产品质量建设有效推进，“三资”投入农业力度加大，乡镇工业销售收入增长30.4%。服务业克服非典的负面影响，实现增加值333.8亿元，增长13.3%。旅游总收入增长25.7%，10个重点商品市场交易额增长21.5%。存贷款余额分别达到1170.6亿元和799.1亿元，增长29.4%和42.7%。同时，房地产市场活跃，信息服务业比重上升。

——开放型经济取得新成绩。利用外资规模不断扩大，全年新批协议利用外资20.5亿美元，增长68.2%，实际利用外资8.55亿美元，增长52.4%。对外贸易大幅增长，实现进出口总额52.5亿美元，增长34%，其中出口达到35.3亿美元，增长32.9%。外经营业额达1.2亿美元。沿江开发有了新的进展，编制完成沿江发展规划及沿江启动区控制性详细规划，实施录安洲开发、通用码头二期工程等重大基础设施项目，江边精细化工区建设拉开序幕。国家级一类口岸、高新区海关“直通点”正式运行。各级各类园区建设成效显著，专业化功能日趋明显，钟楼开发区后来居上，重点乡镇工业园区建设快速推进。成功地举办了城建服务业招商会和第八届中国国际中小企业商品博览会。

　　——改革转制实现新突破。国企改革取得重大进展，完成了市属国有、集体企业改制任务，基本实现企业股权多元化。加大投融资体制改革力度，组建了城市建设集团、交通产业集团等政府投资主体；民间投资空前活跃，占全社会固定资产投资的比重达59.7%。私营个体经济快速发展，私营企业和个体工商户数分别超过3万户和10万户，注册资本突破300亿元，从业人员超过60万人，实现增加值358.8亿元，增长45.8%。加快转变政府职能，深化行政审批制度改革，率先推行企业登记注册告知承诺制，全面实行部门预算制度。首批24家生产经营性事业单位改制任务顺利完成。农村税费改革继续深化，农民实惠得到进一步增加。

　　——城市功能得到新提升。经过全市上下的不懈努力，我市被命名为国家卫生城市，并通过环保模范城市国家级验收。金坛、溧阳同时被命名为国家卫生城市。生态环境进一步改善，荣获“中国人居环境范例奖”。完成新一轮城市总体规划的编制论证，完成开发园区和沿路、沿河、沿景观等专项规划及地形图航测。规划的决策、执行和监督机制进一步完善，成立了规划委员会及其规划审议鉴定委员会和专家顾问咨询委员会，重点规划设计面向国内外招标。基础设施力度加大，交通、市政等建设投入创历史最高水平。大交通建设快速推进，锡宜高速常州段、宁杭高速溧阳先导段建成通车，沪宁高速常州段扩容和常宁、扬溧高速常州段开工建设，在建高速公路里程达到220公里，全长110公里的常州大外环全线竣工。建成了一批网络型、功能型市政设施，春江路和龙江路北段、青洋路北段、勤业西路、武进长江引水一期、魏村水厂、戚墅堰污水处理厂一期、市河整治等重点工程相继竣工，拓宽改造了兰陵路、清凉路、采菱路等市区道路。成功地承办了第三届江苏省园艺博览会，建成南市河滨河绿地、五星公园、怀德广场等一批绿化景点，完成淹城遗址森林公园一期工程，东坡公园扩建改造，芦墅公园对外敞开，新增城市绿地239.5公顷。开展了城市管理相对集中行政处罚权试点工作，市容市貌和市区交通的专项管理取得实效。进一步加强土地资源的配置和开发利用，强化政府对土地一级市场的垄断管理，土地出让收益大幅增长。与此同时，顺利实施了市区部分乡镇、街道的行政区划调整，社区建设稳步推进，城乡一体化步伐明显加快。

　　——人民生活水平有了新提高。城镇居民人均可支配收入达11307元，农民人均纯收入5550元，分别增长13.8%和8%，全市人均储蓄17992元，比上年增长23.5%，全社会消费品零售总额达280.7亿元，增长12.8%。就业和再就业工作得到加强。全年净增城镇就业人数10.37万人，1.3万名“4050”人员和双下岗失业人员实现再就业。城镇登记失业率控制在3.5%以内。社会保障工作积极推进，企业养老保险和医疗保险参保人数分别净增4.18万人和8.86万人。群众居住条件不断改善，200万平方米的9个老住宅小区整治任务提前完成，200户“双特困”家庭入住廉租房，经济适用房蓝天小区开工建设，全年市区发放住房公积金个人贷款6.33亿元。

　　——社会事业获得新发展。深入实施科教兴市战略,科技对工业和农业增长的贡献率分别达44%和56.4%。获准建设我市第一个国家级工程技术研究中心和4个特色产业基地，新增省级高新技术企业33家、高新技术产品125只，全市专利申请量达1500件，再次通过全国科技进步先进城市考核。常州大学城一期工程顺利竣工，首批1.4万名学生如期入学；高中入学率达到96%，全市高考万人进线率连续6年居全省第一。大力实施人才强市战略，全年引进各类人才首次突破1万名。文化广电事业取得新成绩。编制完成《常州市历史文化名城保护规划》，修复西瀛里明城墙，电视剧《天娇》等14件作品分别获得全国、省“五个一工程”奖，总成绩名列全省第二。数字电视在全省率先实现辖市区开通。医疗卫生水平进一步提高，一批临床医疗技术居全省领先地位，完成卫生监督和疾病预防控制体制改革，公共卫生体系初步建成。成功地举办了市第十二届运动会和第六届全国残运会常州赛区的赛事，连续七年被评为全民健身全国先进城市。人口与计划生育工作水平不断提高，人口自然增长进入零增长。与此同时，学习型城市建设深入推进，群众性精神文明创建活动蓬勃开展，连续三次获得“全国双拥模范城”称号。依法治市和民主法制建设进一步加强，廉政建设和反腐败斗争向纵深推进。新闻出版、地方志、档案、哲学社会科学、防震减灾、民族宗教等工作深入开展，台湾事务、侨务、外事工作和国防建设取得长足进步，民政、妇女儿童、老龄、关心下一代等各项事业也都取得了新的成绩。

　　各位代表，过去的一年，我们遇到的困难比预想的多，取得的成绩比预想的好，经济社会各个方面都在发生新的积极的变化。成绩来之不易，这是全市人民在中共常州市委的领导下，团结奋斗、锐意进取的结果，是人大、政协监督支持的结果。在此，我代表市人民政府，向全市人民，向人大代表、政协委员、各民主党派、工商联、无党派和各界人士，向驻常部队指战员和武警官兵，向所有关心、支持常州建设和发展的海内外朋友们，表示衷心的感谢！

　　回顾总结一年来的工作，我们主要有以下几点体会：

　　（一）坚持以“两个率先”为目标，进一步明确工作思路。我们以发展为第一要务，以“两个率先”的宏伟目标统一思想、凝聚人心、激励斗志，坚持审时度势，扬长避短，乘势而上。在深入开展调查研究的基础上，认真思考常州的过去、现在和未来，提出了扩大县域经济总量、提升新型工业化和经济国际化水平、加快服务业发展等符合常州实际的经济工作举措。在实际工作中，我们坚持以发展的新思路谋划新优势，以改革的新突破增创新优势，以开放的新局面扩大新优势，以各项工作的新举措强化新优势。

　　（二）坚持转变职能，着力提高行政水平。我们始终坚持有所为有所不为，对可以由市场机制调节的领域坚决不为，努力从繁杂的事务中解脱出来；对规划调控、资源配置、培育主体、制定政策、依法监管等方面力求有为，集中精力议大事、谋大事、办大事。我们强化了规划的地位和作用，加强对城市资源特别是土地资源的配置与管理，组建了适应城市建设需求的投融资主体，研究出台了促进民营经济、规模经济、开放型经济发展和科技进步的政策措施，并大力整顿和规范市场经济秩序，切实加强安全生产监督管理，有力地推动了经济社会的快速协调发展。

　　（三）坚持以重点带动全面，不断创新工作方法。一年来，我们着力抓好关系经济和社会发展全局的大事，统筹安排，果断决策，举全市之力，积极推进。对年初确定的国企改革、园区开发、城市建设和民营经济发展等重点工作，我们一着不让，想方设法，确保目标任务全面完成。对事关常州长远发展的沿江开发、大交通建设中的高速公路骨架、运河南移、农村道路、312国道改线等重大基础设施项目，以及投融资体制改革、经济结构调整和规模经济发展等等，我们精心谋划，落实措施，着力构建加快发展的新平台，努力形成自身特色和优势，为增强常州综合竞争力奠定坚实的基础。

　　（四）坚持亲民务实，切实改进工作作风。一年来，我们坚持以民为本，始终把人民的利益放在首位。面对突如其来的非典疫情，我们沉着应对，快速反应，迅速形成了全民动员、全面预防、覆盖城乡的防治体系，成功创建了非典防治安全区。我们制定出台了多项鼓励和促进就业创业的政策措施，探索建立农村基本养老保险制度，符合“低保”条件的实现了“应保尽保”。年初确定的为民办实事项目全面完成，“农村五件实事”紧张有序推进。按照“抓紧每一天、走好每一步、干好每一年”的要求，突出重点，坚持市政府常务会议和市长办公会议制度，及时研究解决经济社会发展中的重大问题；建立了开放型经济例会制度，及时协调处理发展中的矛盾和问题，并强化督查，狠抓落实，树立勤政务实的政府形象。

　　在充分肯定成绩的同时，我们必须清醒地看到工作中存在的问题和前进中面临的困难。主要是：经济结构性矛盾仍然突出，推动经济快速发展的大企业、大项目偏少，利用外资与先进地区相比还有较大差距；市场取向改革仍需进一步拓展，国有资产管理体制有待完善；城市基础设施建设任务繁重，环境保护尚需加强，城市长效管理机制还需进一步健全；城乡经济发展不够平衡，农民增收渠道不多；就业和再就业压力较大，城乡社会保障体系需要进一步完善。同时，创业创新的环境需要不断优化，政府机关及公务员的发展理念、工作作风和服务水平有待进一步改进和提高。对这些问题，我们将高度重视，并在今后的工作中认真加以解决。

　　二、2004年主要目标任务

　　2004年是贯彻落实党的十六届三中全会精神、全面推进改革开放、加快“两个率先”的重要一年，也是完成“十五”计划的关键一年。综观国内外宏观环境，世界经济逐步回升，国际制造业向“长三角”地区转移的趋势依然强劲；我国正处于经济发展的重要战略机遇期，扩大内需的累积效应和巨大市场潜力进一步释放，特别是十六届三中全会作出的决定，将极大地改善经济发展的体制环境。从我们自身实际来看，力争“两个率先”、实现富民强市已成为全市人民的共同目标和自觉行动，经济社会发展的增长动力、机制活力和发展后劲日趋显现，“勤学习、重诚信、敢拼搏、勇创业”的市民精神，正在成为我市实现跨越式发展的强大精神动力。这些都为我们做好今年工作奠定了坚实的基础。但是，我们也要清醒地看到，世界经济存在不确定因素，我市经济社会生活中还有一些亟待解决的突出问题，常州面临的竞争更加激烈，经济社会进入了一个十分关键的发展阶段。我们必须进一步解放思想，更新观念，树立强烈的机遇意识、责任意识、竞争意识，实现发展理念和发展思路的与时俱进，只争朝夕，求真务实，全力夺取现代化建设事业的新胜利。

　　今年政府工作的总体要求是：以邓小平理论和“三个代表”重要思想为指导，全面贯彻党的十六大、十六届三中全会精神，树立和落实科学的发展观，紧紧围绕“两个率先”的目标，进一步解放思想，抓住机遇，保护、引导和发挥好各方面的积极性，坚持以加快富民为核心、改革开放为动力、结构调整为主线，加快体制机制创新，加快新型工业化和现代制造业基地建设，加快沿江开发和各类园区建设，加快城市化和城市现代化，加快富裕农民和提升农业、农村经济发展水平，着力推进经济增长方式的转变，提高增长的质量和效益，保持经济快速稳定增长的势头，促进社会事业繁荣进步，实现经济社会全面、协调、可持续发展。

　　2004年国民经济和社会发展的主要预期目标是：国内生产总值突破1000亿元，增长13%，力争增长15%；财政收入增长15%，力争增长18%以上；全社会固定资产投资增长50%，外贸出口增长20%，协议注册外资增长50%，实际利用外资增长50%，全社会消费品零售总额增长13%，城镇居民人均可支配收入增长13%，农民人均纯收入增长8%，城镇登记失业率控制在4.5%以内，人口自然增长率控制在1‰以内。

　　为完成上述目标，我们将重点抓好以下几项工作：

　　（一）提高经济国际化水平，力争开放型经济迈上新台阶

　　牢牢把握国际生产要素和产业转移带来的新机遇，按照“突出外资、提升外贸、扩大外经”的总体思路，大力提高全市开放、全面开放、全方位开放的水平。

　　集中力量加快沿江开发，推进开发园区建设。全面实施沿江开发发展规划，重点抓好录安洲夹江大桥及万吨级码头、西绕城高速公路及通用码头二期工程建设。按照“明确功能定位、塑造特色优势”的要求，进一步加快开发园区的建设和发展，完善基础设施，增强专业化功能。在大力发展劳动力密集和加工型产业的同时，更加重视发展资本、技术密集型产业。积极探索市场开发、联合开发的路子，优化配置和集约利用各类资源，加快省级以上开发区的标准厂房建设，提高土地利用率和投资强度。进一步形成壮大电子、环保、纺织、精细化工和现代工程机械等专业园区，发挥产业集群作用和规模效益。

　　创新招商机制，在引进大项目上求突破。进一步创新园区管理体制和机制，提高服务水平，更新招商理念，依托制造业基础，发挥民营经济快速成长优势，找准外资与我市产业的结合点，拓展项目源，培植新项目。创新招商方式，突出专业招商、资源招商、委托招商，积极推进以外引外、以商引商。强化招商队伍建设，完善招商激励机制，形成市、辖市区、各类园区的招商网络体系。加强与日韩、欧美、台湾等重点国家和地区的大公司、大集团的合资合作，在引进基地型、龙头型、科技型等超亿美元大项目上取得新突破。在招商引资的同时，大力引进先进技术、管理经验和高素质人才。继续办好中国国际中小企业商品博览会。

　　全力推动外贸外经持续增长，积极开展区域合作。坚持以质取胜、科技兴贸和市场多元化战略，积极发展加工贸易，优化出口商品结构，确保机电产品、高新技术产品的出口比重分别达到50%和15%以上。主动适应出口退税政策的调整，加强税贸、关贸、银贸协作和相关部门的协调配合，进一步壮大外贸主体。继续实施“走出去”战略，发展海外经济。扩大与港澳地区的经贸往来，加强与上海等“长三角”城市的交流合作，继续做好外事、侨务、口岸办和台湾事务工作。

　　（二）紧紧围绕农民增收，全面发展农业和农村经济

　　针对当前农村工作的新形势、农业生产的新情况、农民增收的新矛盾，更加自觉地把解决好“三农”问题作为经济工作的重中之重，坚持统筹城乡经济和社会发展，进一步加强农业、支持农业、保护农业，千方百计增加农民收入。

　　加快农业经济结构调整。积极实施《常州市现代农业产业发展总体规划》，在保护和稳定提高粮食生产能力的基础上，不断壮大特色优势产业规模，力争花卉苗木面积达20万亩，特种水产覆盖率达到95%，加快形成优质时令水果产业带和畜禽规模养殖基地。大力推进农产品品牌建设，加快农业产业化经营，着力培育龙头企业、专业市场和合作经济组织。进一步吸引“三资”投资开发农业，大力扶持发展农产品加工业和出口创汇农业。加快农业科技创新和社会化服务体系建设，加强农业生态环境建设，继续搞好农产品质量标准、检验检测、市场准入等制度建设，从源头上保证农产品质量安全。强化农产品现代营销体系建设，办好2004年常州名特优农产品上海展销会。

　　切实抓好非农产业发展和农村改革。充分发挥乡镇工业园区的带动作用，大力发展乡镇工业，培育壮大建筑产业，推进农村商贸等第三产业的发展，努力增加农民的工资性、经营性收入。加强农村职业技术培训，提高农民素质和农村劳动力转移的组织化水平，确保全年转移农村劳动力2万人。加快农村土地使用权的有序流转，积极稳妥地发展规模经营。进一步完善土地征用制度，建立失地农民社会保障机制。积极推进农村集体经济股份制改革，探索增加农民收入的新路子。

　　进一步优化县域经济发展的基础条件。加大县域基础设施建设投入力度，基本完成“农村五件实事”，切实改善茅山老区的生产、生活条件。抓好农村水利建设，继续搞好太湖治理、长江堤防达标等流域重点工程，开工建设武宜运河拓浚工程和澡港河南段整治工程。改造建设农村公路1500公里，形成市、辖市、镇、村的等级道路网络。扬长避短，搞好规划布局，拓展发展空间，发挥资源优势，努力提升县域经济水平。

　　（三）调整优化经济结构，坚持走新型工业化道路

　　抓住我国正进入新一轮经济增长周期的有利时机，按照新型工业化的要求，加快结构调整，努力实现速度、质量、效益的协调和统一，提高工业经济竞争力。

　　加快产业结构优化升级。充分利用世界制造业加快调整的机遇，高起点推进制造业基地建设，重点运用高新技术和先进适用技术改造提升传统产业，做大做强机电产业，培育壮大电子信息、生物医药、新材料、精细化工等产业。促进工业科技进步，推进软件园等特色产业基地建设，新建4家省级以上各类工程技术研究中心和开发中心，实施50项制造业信息化示范项目，新增省级高新技术企业20家、高新技术产品100只，努力开发和保护一批具有自主知识产权的新产品。

　　多渠道增加工业投入。在充分考虑环境、资源承载能力的基础上，大力利用海外资本和金融资本，激活民间资本，积极争取国债项目和各类政策性扶持资金，形成多元化的投入渠道。落实积极的扶持政策，狠抓重点产业、重点企业、重点项目的投入，确保工业投入350亿元以上，加快85个工业超亿元项目的建设进度，力争65个新增长点项目实现销售收入超200亿元。

　　加快大企业大集团建设。发挥60家重点企业的“领头羊”作用，推进规模经济发展，为建设现代化特大城市提供有力的支撑。坚持市场主导与行政推动相结合，通过体制机制创新、资本扩张、联合重组、科技进步、品牌建设、外向开拓等措施，促进优质资源向支柱产业、优势企业和名牌产品集聚。搞好资金、能源等生产要素的组织协调，做好企业上市工作，更好地为大企业大集团服务。年内力争有两家企业销售收入达到100亿元，一批重点企业销售收入分别超10亿元、20亿元和50亿元。在发展规模经济的同时，进一步健全中小企业服务体系，促进面广量大的中小企业快速健康发展。

　　（四）培育扩大内需，加快发展现代服务业

　　把加快发展服务业作为扩大就业、优化产业结构和促进经济社会协调发展的重要举措，在扩大规模、拓展领域、提高水平上下功夫。

　　大力提升商贸流通业。积极拓展商贸流通领域，大力发展连锁经营、加盟经营、品牌经营、电子商务等新型业态，积极吸引大型商贸企业进入我市。放大餐饮业特色优势，加快建设南大街商业步行街，开工建设西瀛里商贸中心，做好武进区、新北区等商贸中心的规划和启动工作。加强市场建设，培育红星集团等十大品牌企业、夏溪花木市场等十大重点市场和江苏湖塘纺织品交易中心等十大重点项目。加快综合交通运输体系建设，启动建设新北区物流园区和凌家塘物流中心，大力发展第三方物流，逐步形成区域性物流基地。

　　加快发展旅游业。积极实施常州市旅游发展规划及辖市和重点旅游景区规划，开展古运河风光带专项规划的前期调研论证工作。着力推进恐龙园、太湖湾等六大旅游景区综合开发，全面完成天宁宝塔等重点项目建设，形成常州旅游的新优势。加强以龙文化为主线的旅游形象宣传，举办好中华龙城（常州）旅游节，提升常州旅游整体形象，积极拓展国内外旅游市场，全年实现旅游总收入增长17%以上。

　　培育壮大金融业。进一步提高金融业发展和金融安全水平，促进金融与经济良性互动发展，确保各项存款和贷款比年初分别增加250亿元和200亿元。积极争取市信托投资公司重新登记，促进东海证券在上市通道、国外合作、投资银行业务上迈出新步伐。支持国内外资本参股常州商业银行，吸引各类商业银行和保险公司来我市设立分支机构。与此同时，规范发展律师、会计等社会中介组织，加快提升信息服务和社区服务业。

　　进一步扩大消费需求。消费需求是最终需求，是拉动常州经济增长的根本动力。要抓住住房、汽车、旅游、信息服务和非义务教育等消费热点升温的有利时机，带动一批产业的发展。进一步规范房地产市场，增加普通商品房和经济适用房供应，引导和促进房地产业健康发展。扩大城乡消费市场，完善消费政策，健全个人消费信贷体系。加强市场管理和监督，优化消费环境，使城乡居民放心消费。

　　（五）大力推进体制机制创新，增强经济社会发展活力

　　按照十六届三中全会决定的要求，坚持整体推进和重点突破相结合，围绕所有制结构调整和现代产权制度建设，积极推进各项改革，建立更具活力、更加开放、更有效率的市场经济运行体系。

　　巩固和扩大国有企业改革成果。按照“归属清晰、权责明确、保护严格、流转顺畅”的要求，完善各类资本有进有退、合理流动的机制，大力发展产权多元化的混合所有制经济。认真解决空壳企业处置等改制过程中的遗留问题，引导已改制企业转换经营机制，完善法人治理结构，坚持体制、机制和管理同步创新，加快建立现代企业制度。进一步理顺国有资产管理体制，探索建立国有资产授权经营制度，完善国有资产监管和考核体系。

　　加快事业单位及公用行业改革。按照政事分开、分类指导的原则，对行政管理类事业单位，进行清理和规范；对社会公益类事业单位，调整结构布局，优化资源配置，增加政府投入，提高服务水平；对生产经营类事业单位，按照市场化、社会化、产业化方向，加快改制转企步伐。推进城市公用事业改革，放开市场、打破垄断、引入竞争，鼓励外资和社会资本进入，逐步建立适应市场经济要求的市场竞争机制、企业经营机制和政府监管机制。

　　放手发展民营经济。实现富民强市，必须充分激活民间资本，加快发展民营经济。大力鼓励创业致富，鼓励民众创业、自主创业、艰苦创业，营造“想创业、敢创业、会创业、创大业”的浓厚氛围，真正让民间资本、技术和人才等各类要素的活力竟相迸发，让民众创造财富的源泉充分涌流。进一步拓展发展空间，彻底清理和修改限制非公有制经济发展的政策和法规，放宽市场准入，凡是法律法规没有禁止的领域，凡是对外资开放的领域，凡是国有、集体资本退出的领域，都要对民资开放。选择一批经营性政府投资项目，采取转让经营权、股权等形式向民间资本开放，支持有条件的民营企业通过股份制改造、上市融资、债券发行等多种形式筹措发展资金，支持科技型、外向型、规模型民营企业加快发展。同时，加强社会信用体系建设，促进民营企业提高经营管理水平，切实维护职工的合法权益。

　　加强就业和社会保障工作。坚持劳动者自主择业、市场调节就业和政府促进就业的方针，继续实施积极的就业政策，重点援助“4050”和双下岗失业人员实现再就业。加快建立“老有所养、病有所医、贫有所助、居有所屋”的社会保障体系，提高社会养老保险、医疗保险的覆盖率和基金的征缴率，提高住房公积金的覆盖面和汇缴率；发展慈善事业，关爱困难群体，完善城乡居民最低生活保障制度，多渠道、多形式对低收入居民实施社会救助。

　　（六）加强城市建设和管理，推进城市化和城市现代化进程

　　继续围绕构筑区位优势、增强城市功能、营造城市环境和美化城市形象等重点工作，加快推进城市化和城市现代化进程。

　　完善城市功能定位。按照构建“一体两翼”特大城市框架和“提升中心、拓展南北、完善东西”的要求，在新一轮城市总体规划的基础上，抓紧编制、完善中心组团等分区规划和专项规划。加强对城区“农夹居”、“城中村”整治和老企业搬迁的规划工作，抓紧编制新运河两侧概念性规划和轨道交通规划，不断提升中心城区形象，增强中心城区辐射集聚功能；按照“高新技术示范区和现代化城市新城区”的要求，完善新北区的产业定位和功能分区布局；依托大学城和淹城遗址、滆湖、太湖等资源，按照“现代科教区、高新技术集聚区和生态休闲居住区”的要求，加快武进区一批专项规划的编制；进一步提高天宁、钟楼、戚墅堰区控制性详细规划的覆盖率，加快区级城市副中心的建设步伐。金坛、溧阳要按照具有山水特色的现代化中等城市的要求，搞好空间布局、市政设施、生态保护等规划；抓紧搞好小城镇特别是重点中心镇的规划建设。

　　构建城市现代化基础设施体系。为高质量、高水平发挥城市综合服务功能和集聚辐射作用，着力实施大交通战略，建成宁杭高速、122省道常州段和金武线等工程，加快常澄高速、常宁高速、扬溧高速、沪宁高速扩容、西绕城高速、运河南移、312国道改线和239省道等工程建设。加速城市路网建设，大力推进常澄路-万福路等道路拓宽改造工程，开工建设春江路、青洋路、龙江路南段和新岱路北段，拓宽丽华路、常焦路、晋陵南路、常戚路等城市干道，改造延陵西路、大仓路—芦墅路—飞龙路、关河东路、吊桥路等道路。加快建设江边污水处理厂一期、生活垃圾卫生填埋场二期、市南和戚区两个公交中心、垃圾焚烧发电和天然气利用等市政公用设施项目，积极实施城市防洪工程，加快城市人防设施建设。做好常泰通道建设的前期准备工作。

　　加强生态环境建设和管理创新工作。着力提高城市绿化覆盖率，不断提升绿化品位，推进“绿色常州”建设。加大水环境整治力度，继续抓好武进港、太滆运河和市河综合整治工作。发展循环经济，从调整产业布局和结构着手，控制减少工业污染源，推进环境建设再上一个新台阶。深化“两级政府、三级管理”的城市管理体制改革，完善城市管理综合执法体系，加大规划执法管理力度。加快编制各级各类减灾预案和应急保障计划，增强处置能力。充分发挥市属投资主体的作用，进一步提高城市各类资源的市场化运作水平，完善经营性用地使用权的出让程序，注重特许经营权等无形资产的开发利用。

　　（七）加快发展社会事业，促进“三个文明”协调发展

　　社会事业的繁荣和发展，是全面建设小康社会的内在要求。要继续实施科教兴市战略，加快发展各项社会事业，进一步增强可持续发展的能力，促进经济、社会和人的全面发展。

　　加强科技创新和人才队伍建设。深化科技体制改革，强化科技创新体系建设，加大科技投入，促进科技资源有效综合集成。推动高新园区“二次创业”，加快高新技术特色产业基地建设，重点推进制造业信息化、新药、新材料三大科技示范工程建设，增强技术创新能力。促进产学研合作和科研成果转化，实施40项市级重点科技攻关项目。大力推进城市信息化，努力建设“数字常州”。强化人才强市观念，优化人力资源配置，完善引进和使用高层次人才和紧缺专门人才的激励机制，积极营造有利于人才施展才华的环境。

　　推进文教卫体事业快速发展。调整教育布局和结构，优化教育资源配置，不断深化教学改革，推进教育体制和机制创新，大力实施素质教育。开工建设常州大学城二期工程，努力提升高等教育的规模和水平。建立和完善以县为主的农村义务教育管理体制，进一步加强和发展农村教育。完善和规范以政府投入为主、多渠道筹措经费的教育投入机制。实施“蓝天计划”，努力解决流动少年儿童的入学问题。加快文化体制改革，积极探索文化产业化的新途径。加强文化设施建设和文物保护，重点建设博物馆新馆等文化设施，启动修缮前后北岸历史文化保护街区，修复意园、黄仲则故居。深化医药卫生体制改革。加强疾病预防和公共卫生监督，提高公共卫生服务水平，增强突发公共卫生事件的应急处理能力。开工建设中医院综合楼和新北医院等一批重点项目。不断完善计划生育利益导向机制和服务体系，稳定低生育水平。加快体育中心二期工程规划设计与开工建设，广泛开展全民健身活动，认真备战第16届省运会，积极申办第17届省运会。切实做好民政、防震减灾、新闻出版、民族宗教等各项工作，积极发展老龄、妇女儿童、关心下一代、残疾人等事业。

　　加强精神文明建设和民主法制建设。以争创全国文明城市为目标，深入开展群众性精神文明创建活动，办好第三届常州艺术节。继续推进学习型城市建设，实践新时期常州市民精神。广泛开展科普教育，大力弘扬科学精神。加强基层政权建设，开展“示范社区”创建活动，推进社区居民自治和农村村民自治。深入开展“四五”普法教育，提高市民的法律素质。进一步拓展和规范法律服务，积极开展法律援助。加强国防动员和民兵预备役建设，搞好“双拥”活动。认真办理人大代表建议和政协委员提案，充分发挥工会、共青团、妇联等人民团体的桥梁和纽带作用，重视并做好群众来信来访工作。深入开展“建设平安常州、创建最安全地区”活动，严厉打击各类犯罪活动，加强社会治安综合治理，高度重视安全生产，切实保障人民群众生命财产安全。

　　（八）切实加强自身建设，不断提高政府工作水平

　　按照有所为有所不为的要求，提高政府调控经济、管理社会、服务人民的能力和水平，建立行为规范、运转协调、公正透明、廉洁高效的行政管理体制，努力建设法治政府、责任政府和服务型政府。

　　坚持高效服务，切实转变政府职能。按照强化、弱化和转化的要求，该管的事一定管住管好，不该管的事坚决不管。进一步深化行政审批制度改革，减少审批事项和行政事业性收费项目，进一步扩大“告知承诺制”的适用范围，建设重大投资项目“绿色通道”，实行年度审检集中办理、联合办理，逐步推行网上审批，提高审批效率。大力改善投资软环境，积极营造奋发有为的舆论环境、优质高效的政务环境、严明规范的法制环境和诚实守诺的信用环境，以环境的优化带动竞争力的提升。

　　坚持依法行政，切实规范政府行为。以贯彻实施《行政许可法》为契机，严格按照法定权限和程序，履行职责，行使权力，把政府各项行政行为纳入法制化的轨道。推进决策的科学化、民主化、规范化，扩大人民群众对经济社会事务的知情权、建议权、参与权和监督权。进一步完善政务公开制度，加强对行政行为的监督。健全行政执法责任制和评议考核制，提高依法行政的水平。

　　坚持从严治政，切实加强廉政建设。深入开展学习型机关创建活动，大力提高公务员队伍整体素质。认真执行廉洁从政的各项规定，进一步深化纠风工作和专项治理，坚决查处违纪违法案件，着力加强对权力的制约、资金的监管和从政行为的规范，努力从源头上预防和治理腐败。认真实施市区税收属地征管工作，加快推进以部门预算、国库集中收付等为主要内容的财政收支制度改革，逐步建立公共财政体系。加强对建设工程招投标、产权交易、政府采购等行为的管理，深化完善审计监督措施。继续开展效能监察、行风评议和基层评议机关活动。

　　坚持执政为民，切实改进工作作风。树立正确的政绩观，把发展好、维护好、实现好人民群众的根本利益，作为政府工作的出发点和归宿。深怀爱民之心，恪守为民之责，善谋富民之策，多办利民之事，真正做到权为民所用，情为民所系，利为民所谋。力戒形式主义，切实做到思想与时俱进、工作求真务实、作风只争朝夕，扎扎实实地推进各项工作。深入调查研究，善于把上级的方针政策同本地区、本部门的实际结合起来，形成富有成效的工作目标、方法和措施。

　　今年要努力为民办好10件实事：（1）实施农村改水，新增自来水受益人口38万人；（2）全面完成农村3535户草危房改建任务；（3）建设和改造农村道路，实现辖市（区）到镇通二级以上公路、镇与镇通三级以上公路、镇到行政村通四级以上公路；（4）深化农村税费改革，完善农业税收缴办法，加大对茅山老区村级组织和经济薄弱村的资金支持力度；（5）建立完善新型农村合作医疗保险制度，以镇为单位的农村居民参保率达到70%以上，对“五保户”和贫困家庭给予医疗补助；（6）全市城镇新增就业人数3.5万，安置2.5万名城镇人员就业，完成各类再就业培训2.2万人次；（7）增加城市绿地面积，建设青枫公园、新市路滨河绿地、淹城森林公园二期等一批绿化工程，敞开扩建荆川公园；（8）改善居民生活条件，继续对红梅新村等28个老住宅小区共250万平方米实施整治，改造危旧房1500户，建设经济适用房蓝天小区和陈渡桥小区，解决150户“双特困”家庭和直管公房“劈户”的住房问题，完成13万户有线电视网络双向改造，新建100个全民健身点；（9）建成区范围内的公共旱厕全部改造成水冲式公厕；（10）完成西新桥新村、清潭五村、工人新村、翠竹新村、西园村等部分地区的除涝工程。

　　各位代表，回顾过去一年，勤劳智慧的常州人民创造了辉煌的业绩；展望新的征程，“两个率先”的宏伟目标催人奋进。让我们在省委、省政府和市委的领导下，以邓小平理论和“三个代表”重要思想为指导，全面贯彻落实党的十六大和十六届三中全会精神，与时俱进，开拓进取，励精图治，团结奋斗，共同建设更加美好的家园，努力创造更加灿烂的未来！
